# Respiratory Syncityal Virus A and B: three bronchiolitis seasons in a third level hospital in Italy

**DOI:** 10.1186/s13052-019-0704-0

**Published:** 2019-08-28

**Authors:** C. Ciarlitto, A. C. Vittucci, L. Antilici, C. Concato, C. Di Camillo, P. Zangari, A. Villani

**Affiliations:** 1 Academic Department of Pediatrics, Tor Vergata University of Rome, Rome, Italy; 2 Academic Department of Pediatrics, Pediatric and Infectious Disease Unit, Children’s Hospital Bambino Gesù (OPBG), Rome, Italy; 3 Virology Unit, Bambino Gesù Children’s Hospital (OPBG), Rome, Italy; 4 Academic Department of Pediatrics, Research Unit in Congenital and Perinatal Infection, Immune and Infection Disease Division, Children’s Hospital Bambino Gesù (OPBG), Rome, Italy

**Keywords:** Bronchiolitis, Preterm, Respiratory syncytial virus, Hospitalization

## Abstract

**Background:**

Respiratory syncytial virus (RSV) is the main cause of hospitalization for bronchiolitis among infants. RSV is classified into two subtypes, A and B, whose predominance alternates during different epidemic seasons. The clinical impact of viral factors is controversial and many evidences suggest a critical role for the immune host response. Premature children are at the highest risk for severe RSV infection. The main aim of this study is to identify the different RSV subtypes circulating in the last three epidemic seasons and to evaluate whether any of them was associated with poor prognosis in term and preterm infants.

**Methods:**

We performed a retrospective analysis of medical records for all patients aged less than one year which were hospitalized during the winter season between November 2015 and April 2018 with clinical diagnosis of bronchiolitis and nasopharyngeal aspirates positive for RSV.

**Results:**

We enrolled 422 children, of which 50 were born preterm. During the analysis period, we observed a significant increase in the rates of oxygen supplementation and admission to intensive care unit. The evidence shows an alternating pattern in the prevalence of RSV subtypes among term born; in each epidemic season, the prevalent serotype is the cause of the majority of the cases requiring intensive care. Conversely, RSV-A is always prevalent in preterm children and caused most of the cases requiring intensive care.

**Conclusions:**

During the 3 seasons analyzed, we observed an alternating prevalence of RSV A and B. While there are no differences in severity between RSV A and B in term population, RSV-A is prevalent and causes most of the severe cases in the premature group. Furthermore, an increase in RSV-related oxygen therapy and PICU admission has been documented not only in the premature population. Considering the absence of appropriate therapeutic strategies, our work emphasizes the importance of implementing prophylaxis measures against RSV and highlights the urgent need to gain knowledge about immune response to the virus, also in premature children.

**Electronic supplementary material:**

The online version of this article (10.1186/s13052-019-0704-0) contains supplementary material, which is available to authorized users.

## Background

Respiratory syncytial virus (RSV) is the main cause of respiratory infections and of hospitalization for bronchiolitis among infants < 12 months [1].

Globally, RSV causes nearly 34 million lower respiratory tract infections (LRTI) and 3.4 million hospitalizations per year in infants and children < 5 years of age, with an estimated annual increase of 10% [2, 3]. In many countries, including Italy, RSV currently represents a public health issue [4].

RSV was formerly considered a subfamily of Paramixoviridae but was recently reclassified as belonging to the Pneumoviridae family [5]; the virus is characterized by a large envelope and negative-sense RNA, coding for 11 glycoproteins. Two main proteins of the lipidic envelope, the fusion protein (F) and the G protein, are antigenically significant, thus inducing neutralizing antibody response. The F protein is highly conserved, while the carboxy-terminal portion of hypervariable region of G protein is subject to rapid mutations. Considering the different in vitro reaction to monoclonal antibodies due to the virion external G protein, RSV has been classified into two subtypes, A and B [6, 7]. These two major serotypes can simultaneously circulate during epidemic season, but, usually, one prevails over the other. Recently, RSV genome sequencing has enabled the identification of multiple genotypes and to date, the analysis of nucleotide sequences of G protein led to describe 11 RSV-A and 23 RSV-B genotypes [8].

Many studies have described different RSV genotypes and their clinical relevance, also in Italy [9, 10]. Monitoring genotypic characterization of the RSV virus could help in understanding the pathogenesis of the infection, in predicting the clinical severity of the disease, and in selecting candidate strains for development of therapeutic and vaccine strategies. It is recognized by many studies that the RSV infection has a more severe course in premature and immunocompromised children [11–13]. It is clear that both the characteristics of the host (age, prematurity, comorbidity, immunodeficiency) and of the virus (including the subtype) influence the clinical course of infection, however the actual contribution of each factor is still unclear. To date, the clinical impact of viral factors related to RSV is still controversial. While many studies report no differences between the two serotypes [8, 9, 14–16], many others relate the worst prognosis to RSV-A [10, 17–20] and some others to RSV-B [21–23]. In 2014 Cangiano et al. [24] highlighted the major role played by RSV in severe bronchiolitis and different rates of RSV related hospitalization in different epidemic season. A review conducted by Kuhdari et al. [4] confirmed the high impact of RSV infection on hospitalization in Italy, especially in children aged less than one year. Recently Midulla et al. [10] described the important influence of specific RSV genotypes on the clinical course of bronchiolitis in a previously healthy and term population. The authors reported deep differences in the demographic characteristics and in the clinical presentation, but not in the necessity and the duration of oxygen (O2) therapy.

It is well know that infants born at < 37 week gestational age (wGA) are at highest risk for severe RSV infection [25]. Prophylaxis with palivizumab (a monoclonal antibody anti-F protein) reduces RSV LRTI in late-moderate preterm population; however, infants are eligible for prophylaxis only according to specific national guidelines. According to the recommendation by the American Academy of Pediatrics, in 2016 the Italian Drug Agency (AIFA) limited the prescription of palivizumab to the group of < 29 wGA and age < 12 months at the beginning of the RSV season [26, 27]. Since then, several evidences in Italy reported an increase in vulnerability of all preterm infants [28–31]. Therefore, in 2017 the AIFA authorized again the use of palivizumab for preterm infants of ≤35wGA aged less than six months at the start of epidemic season [32].

However, the impact of RSV A and B in terms of incidence and severity, both in full term and preterm population, has not yet been investigated.

The main aim of this study is to identify the trend of RSV-A and B in the last three epidemic bronchiolitis seasons in term born and preterm infants; secondly, investigate whether one of them was associated with a worse prognosis, in both populations. Finally we compared three bronchiolitis seasons in terms of length of stay, use of oxygen therapy and need for intensive care.

## Methods

We performed a retrospective study involving patients admitted for RSV bronchiolitis to the Pediatric Academic Department of Bambino Gesù Children’s Hospital in Rome. We reviewed medical records of all patients hospitalized between November 2015 and April 2018 with a clinical diagnosis of bronchiolitis and nasopharyngeal aspirates positive for RSV. According to the Anglo-Saxon definition of bronchiolitis as the first episode of wheezing in the first twelve months of life, only children with less than one year of age were enrolled in the study. We excluded patients with incomplete medical record. We included in the study preterm infants as born < 37 wGA. Data processing was performed with the Microsoft Excel 2018 software. We obtained demographic characteristics of patients (including gestational age, sex and age), length of stay (LOS), medical history during recovery and necessity of O2 therapy administered in pediatric ward as low flow or high flow nasal cannulas. Finally, we evaluated admissions and LOS in pediatric intensive care unit (PICU) due to non-invasive Continuous Positive Airway Pressure (C-PAP) or invasive mechanic ventilation. In this study we selected the presence of hypoxemia as important clinical outcome, with the oxygen saturation detected at the pulso oximeter ≤92% in ambient air and need for O2 supplementation, the duration of O2 therapy and the PICU admission.

The identification of respiratory viruses on nasopharyngeal aspirates was accomplished by Multiplex RT-PCR. Samples were processed, immediately or after storage at − 80 °C, by AllplexTM Respiratory Panel Assays (Seegene, Korea). Nucleic acids were extracted using the STARMag Universal Cartridge kit (Seegene, Korea) on automated Nimbus IV platform. As recommended by the manufacturer, a total of 200 μl per sample were extracted and eluted in 100 μl of elution buffer. For each reaction, 7 μl of DNA/RNA in a final volume of 25 μl were used. Real time PCR was performed on CFX96 (Bio Rad Laboratories, Italy) with respiratory panel assay kit. The panel is made up of 3 mixes which allow the identification of 16 different viruses (Influenza A and B virus, Respiratory syncytial virus A and B, Adenovirus, Enterovirus, Parainfluenza virus 1, 2, 3 and 4, Metapneumovirus, Bocavirus, Rhinovirus, 3 Coronaviruses (NL63, 229E and OC43) and 3 Influenza A subtypes. An internal control was included in each sample to check both extraction efficiency and PCR inhibition. In every run, a negative control was used to monitor carry-over contamination. The results were analyzed automatically using Seegene software (Seegene Viewer V2.0).

For categorical variables, either Χ2 or Fisher exact test were used to test the statistical difference, as appropriate. To evaluate the relationship between the variables, Pearson correlation coefficient was used. Means were compared using Student’s t test. *P*-values of 0.05 were considered statistically significant.

Formal consent is not required for this kind of retrospective study: any personal data was protected accordingly to the Helsinki Declaration and to Italian law. (Legislative Decree of 30 June 2003, n. 196. Code on the protection of personal data).

## Results

### Demographic characteristics

We evaluated medical records of 422 children: 223 (52,8%) were male, 199 (47,2%) were female. The median age at admission was 2 months and 10 days (SD ±1 month and 9 days); medium LOS was 7 days and 20 h (SD ±7 days). The total number of infants admitted for RSV bronchiolitis from November 2015 to March 2018 was 151 in the first season, 101 in the second and 170 in the third one.

In our cohort, 372 patients were born at term and 50 (11,8%) were born at wGA < 37.

Of them 3 patients were wGA < 29 and received prophylaxis with palivizumab; 47 were born between 29 and 36 wGA and one of them (affected by congenital heart disease) received palivizumab.

Moreover, 19 patients (of whom 7 where preterm) had comorbidity: 6 had genetic syndrome (3 Down Syndrome), 9 had congenital heart disease, 2 esophageal atresia, 2 had broncho-pulmonary dysplasia.

### Clinical characteristics

**Fig. 1:**
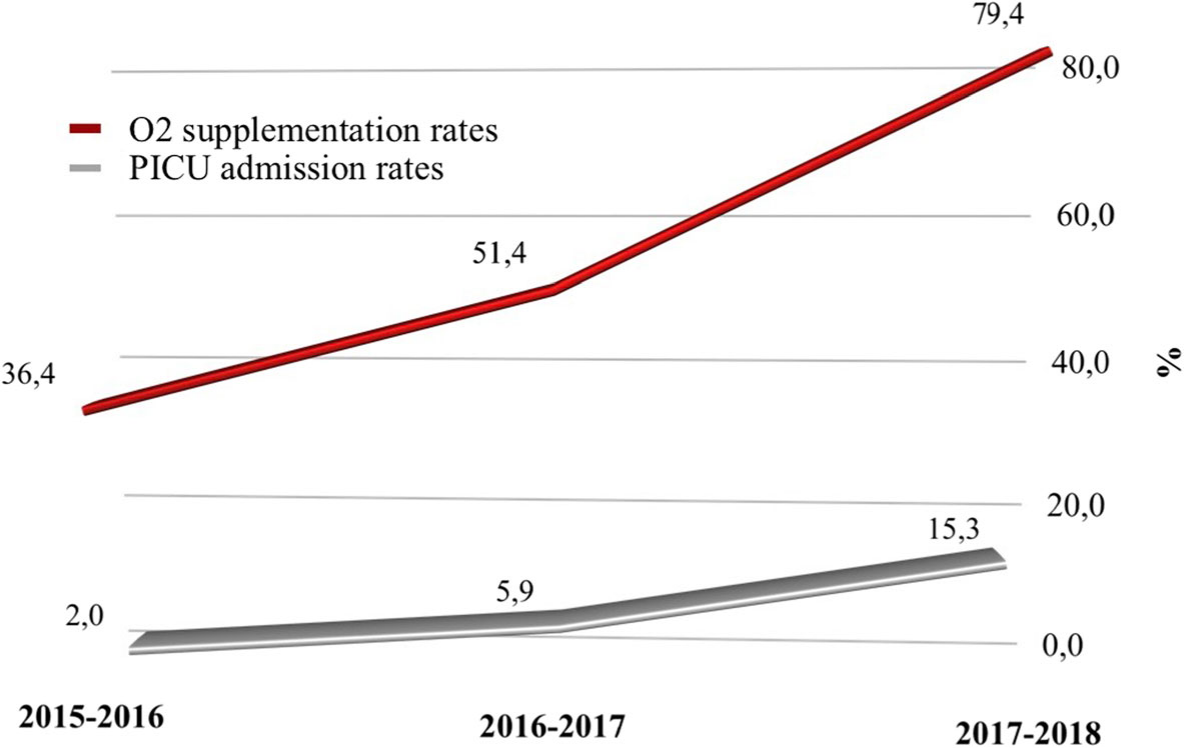
Trend of severity of RSV bronchiolitis from 2015 to 2018. This graphic shows the important increase in rates of oxygen supplementation and admission to Pediatric intensive care unit (PICU) during the entire period analyzed in the whole sample of children admitted to our department

**Fig. 2:**
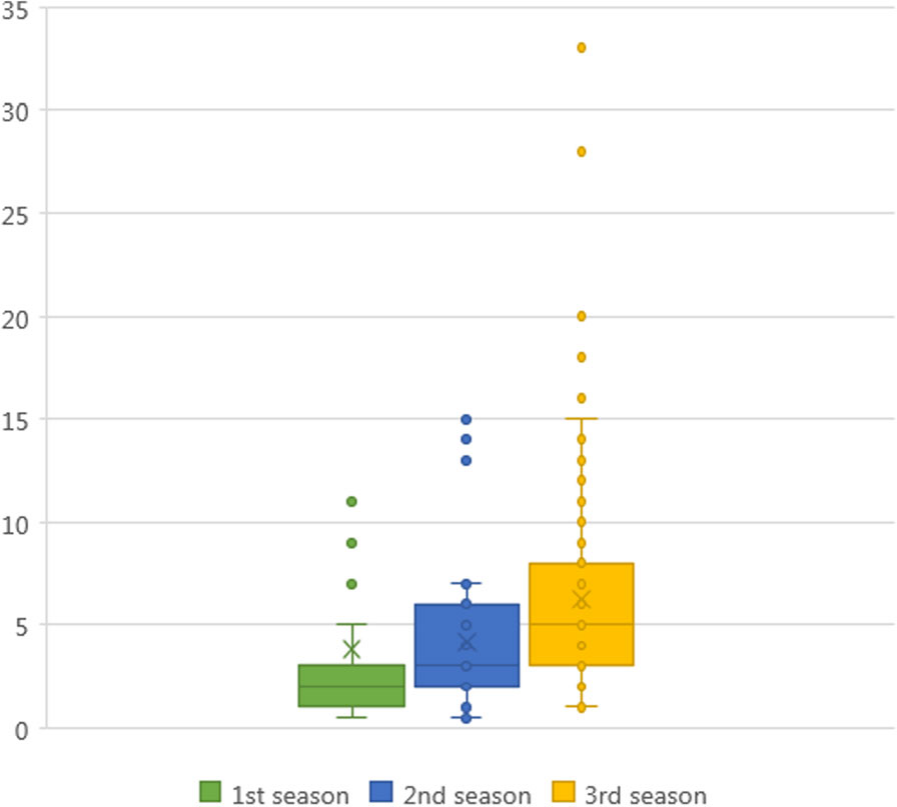
Distribution in days of oxygen therapy in the 3 seasons analyzed

Figure 1 summarizes rates and trend of O2 supplementation and PICU admission among the enrolled infants during the period investigated. The medium duration of O2 therapy was 3,8 days in the first season (2015–2016), 4,2 in the second (2016–2017), and 6.2 in the third (2017–2018) (Fig. 2), with a significant increase between the second and third (*p* < 0.0001).

Among full term born children, 55,9% of infants required O2 supplementation, whereas 8% required PICU admission. The proportion of children that needed O2 therapy increased over the three seasons analyzed: from 35,8% in the 1st, to 47,6% in the 2nd and 78,8%in the 3rd. Comparing the three seasons in progression, we found a significant increase in the need of O2 supplementation (p: < 0.00001). Moreover, this result is independent from the subtype of RSV that caused the infection. Also the percentage of PICU admission increased from 2,19% to 15,2% (p:< 0.0001).

In the premature children population, 62% were treated with O2 therapy in pediatric ward while 10% required PICU admission. During the three seasons examined, the proportion of children treated with O2 increased also in preterm group (42,81%-52,0%-81,21%). We did not have patients requiring PICU in the first season; however, the rate of PICU admission in the second and in the third seasons were respectively 11,8% and 15,8%. Due to the relatively small sample size these results are not statistically significant.

Between the two populations, there was no statistical difference in terms of oxygen supplementation rates (6,71 days ±6 in preterm and 5,02 days ±4,9 in term infants; p: 0,41). Instead, it emerged a strong correlation between wGA and duration of oxygen therapy during admission (Pearson r: − 0.62). Furthermore, the rate of PICU admission for preterm infants was 10% whereas in full term population it was 8%. The mean LOS in PICU was 17,8 days (SD ± 5,93) in premature children, significantly higher (p:< 0,05) compared to LOS of the full term born population (9,83 days SD ±2,64**).**

The comparison of the two populations is summarized in Table 1.

**Table 1:** Comparison of term and preterm infants by clinical and demographic characteristics during RSV bronchiolitis

	PRETERM (*n* = 50)	TERM (*n* = 372)	p value
Demographic characteristics			
Male (%)	50	53.22	0.78
Mean age at admission (± SD, months)	2,5 ± 0,18	2 ± 0,19	0.06
Clinical characteristics			
Mean length of Staying (± SD, days)	9,45 ± 6,93	7,76 ± 7,1	0.11
Rate of O2 therapy (%)	62	55.9	0.16
Duration of 02 therapy (days)	6,71 ± 6,01	5,02 ± 4,92	0.41
Rate of PICU admission (%)	10	8	0.67
Duration of PICU admission (± SD, days)	17,8 ± 5,93	9,83 ± 2,64	0.004

### RSV infection

Among all children, 64,4% had RSV-A, 33,9% had RSV-B, and 1,7% had both RSV-A and RSV-B. During the investigated period, RSV-A was prevalent both in term (64%) and preterm infants (68%). Of the 422 enrolled children, 35,6% had 2 or more virus co-infections and, among them, Rhinovirus was the most represented (19%). Comparing clinical characteristics of patients RSV+ with those infected with RSV and another virus, we found no significant difference in terms of O2 supplementation and PICU admission.

#### Detection of RSV-A and B

Considering the whole population and the entire examined period, we identified an alternating trend in terms of prevalence of RSV-A and B.

The first season (November 2015–March2016) was characterized by RSV-A dominance. Our numbers show a percentage of 90,1% of infections in total of our sample, 90,5% in term infants and 85,7% in preterm infants.

Conversely in the second period (November 2016–March 2017) we found a prevalence of RSV-B (62,3%) in the entire sample with a similar trend in term born infants, where RSV-B accounted for 65,5% of the infections. Overall, also in the preterm population, RSV-A was more prevalent, as we observed 52,9% of RSV-A positivity and 47,1% RSV-B positivity.

During the last winter (November 2017–March 2018) RSV-A emerged again (58,2%) as more frequent, accounting for 57% of the cases in term infants and 68,4% in the premature group.

According to these results, during the overall period, we found no significant differences in the infection rate between RSV A and B within the two populations.

However, our numbers suggest that RSV-A tends to be prevalent in preterm infants (85,7%-52,9%-68,4%), independently from the more represented virus in a given season. Due to the limited sample size, we were not able to prove it statistically. Detailed data are included in additional materials (Additional file 1: Table S1).

#### Clinical course of RSV bronchiolitis in full term and preterm infants

We observed a different clinical impact of the virus within the two population.

In term born infants, the season’s dominant subtype was also responsible for the most severe infection. In the first season, RSV-A accounted for 89,8% of episodes requiring O2 therapy and for 100% of those requiring intensive care.

In the second winter, on the contrary, RSV-B accounted for 72,5% of the cases of O2 supplementation and 100% of PICU admissions. In the third season, among all patients treated with O2, 56,3% were RSV-A positive, accounting for 69,6% of all cases requiring PICU admission.

**Fig. 3:**
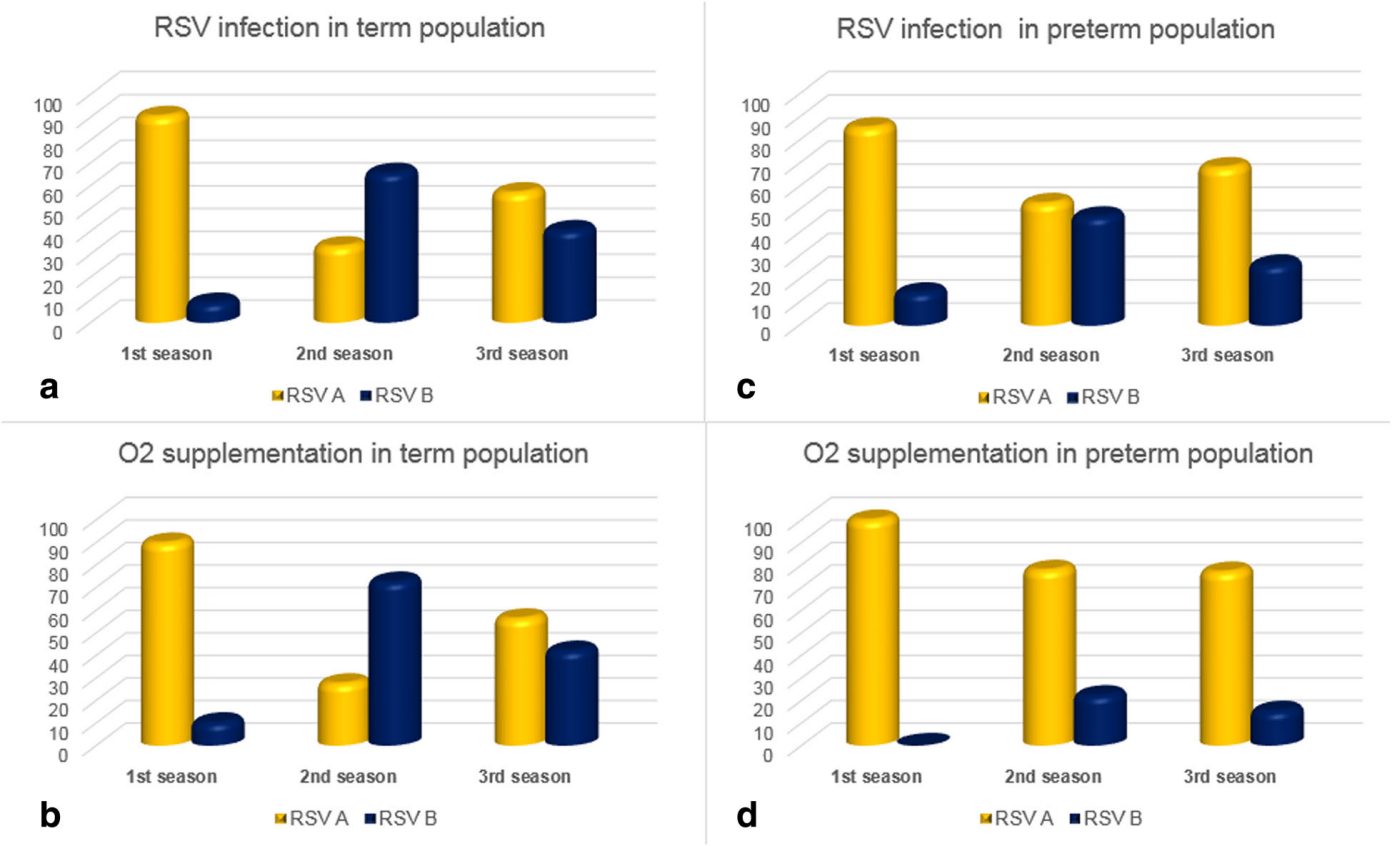
Trend of RSV A vs B infection in full term and preterm infants. In this graphic is shown the different behavior in term of infection and severity of RSV A and B in full term and preterm population. In full term population we observed a seasonal alternation of serotype A and B (**a**); among one season the prevalent serotype is also responsible for most cases requiring oxygen therapy (**b**). In preterm population, RSV A is always prevalent (**c**) and more aggressive (**d**). Numbers are shown in percentage

Based on the chi-square test, we found no statistically significant difference between rates of O2 therapy among term born infants infected with RSV-A and B, neither during the single seasons nor in the 3-year overall analysis. In preterm infants, on the other side, in the first winter all patients treated with O2 therapy were RSV-A +. In the second period analyzed, RSV-A caused 77,7% of cases that needed O2 therapy and all cases admitted to PICU. In the third season, 76,9% of patients requiring O2 therapy and 100% of patients admitted to PICU were RSV-A +. In the overall period, incidence of hypoxemia during RSV-A bronchiolitis is more relevant compared to RSV-B. Unfortunately, the small sample size did not allow statistical analysis. Overall, remarkably RSV-A leads to higher risk of O2 supplementation compared to RSV-B (OR: 2,39) in premature infants. Figure 3 shows the different RSV A and B trend among the two groups in matter of infection and clinical impact.

## Discussion

In the present study we examined epidemiology and clinical characteristics of acute RSV bronchiolitis in term and preterm infants, hospitalized in the Pediatric Academic Department of Bambino Gesù Children’s Hospital in Rome, Italy. We studied 422 patients, of whom 50 were premature with wGA < 37.

The median age at recovery was 2 months with no significant difference between term born and preterm population, while LOS was longer in the group of premature infants.

During the whole reference period (3 years), 56,6% of all patients needed O2 therapy. Furthermore, 8,3% of children were transferred to intensive care unit necessitating C-PAP or ventilatory assistance with orotracheal intubation. We observed an increase in bronchiolitis severity over the three years: a significant increase in the percentage of patients requiring O2 supplementation (*p* < 0.0001) and intensive care cure (*p*: < 0.0001) was reported.

The description of a raising trend in RSV bronchiolitis severity is supported by other recent Italian studies [4, 28, 29], and could be related to the lack of immunity due to the selection of new RSV strains.

Furthermore, in our work, we described a conspicuous number of preterm children admitted to the pediatric department, and in particular of infants included in 29–36 wGA.

Comparing clinical course of bronchiolitis in full term population and in preterm, we found similar percentages of cases requiring O2 therapy. However, duration of oxygen therapy, need for PICU cure and medium of LOS in PICU were more relevant in preterm infants. We found a strong correlation between wGA and duration of oxygen therapy (Pearson r: -0,62). This result confirms the literature data that highlights aggressiveness of RSV in premature infants. [2, 28, 30, 33]. In 2017, a large retrospective work on prematures [13] identified an oxygen supplementation rate of around 80% and a PICU admission rate of 22%, while in our work emerged a lower rate both for O2 supplementation (62%) and PICU admission (16,1%), but a raising trend for both parameters.

During the years 2015–2018 RSV-A was prevalent among all infants enrolled in this study. Although this result is in line with the previous literature [8–10] we studied three consecutive seasons, of which the intermediate was characterized by RSV-B prevalence, and this may be a confounding factor.

Considering the epidemiological trend of the virus, we observed interesting differences between the two age groups analyzed. A seasonal trend of RSV subtypes, which alternate from year to year, occurred in the term born population. In fact, we documented a clear prevalence of RSV-A in the first winter, of RSV-B in the second and again of RSV-A in the third. Conversely, in preterm infants, RSV-A was predominant in all three epidemic seasons. Comparing the severity of RSV bronchiolitis, our data show even more relevant differences between the two groups.

In full term infants, the prevalent serotype among the season was also responsible for most cases requiring oxygen supplementation and intensive care. In preterm infants instead, over the three investigated seasons, the rates of infection by RSV-A were higher compared with those caused by RSV-B, independently from the prevalent circulating serotype. Remarkably, the incidence of desaturation and the use of O2 therapy during RSV-A bronchiolitis was always prevalent compared to RSV-B bronchiolitis. According to this result, in premature infants RSV-A was related to an higher risk of O2 therapy compared to RSV-B (OR 2.39).

Our work, in line with current literature [17–19], describes seasonal alternation of RSV serotypes A and B. This would explain RSV-A prevalence in our sample, since we have analyzed three sequential seasons. In our case series, there is no evidence of a serotype being more aggressive than the other, but we observed a relative increase of severe bronchiolitis for RSV-A, especially for premature children, as already considered by Esposito et al. [9]. Recently, an Italian study [10] described a worse clinical outcome in RSV-A related bronchiolitis, arguing that low weight and younger age at admission were more often associated with novel RSV-A genotype infection. In comparison with their results, we did not find differences in percentages of O2 therapy between RSV A and B, as the prevalent serotype showed a major virulence in general population. However, we found an increased relative risk of O2 therapy in RSV-A infected premature patients. Many studies have investigated the clinical relevance of environmental factors in severe clinical course of RSV infection [12, 34, 35]. In our study, however, the higher incidence of serotype A in preterm population, together with the resultant severe clinical course, suggests a higher virulence of RSV-A per se. This could suggest an important involvement of the immune response to the virus and a role for different immune mechanisms in response to the virus in preterm infants. Indeed, there are many evidences about lack of immunity in preterm children, regarding count and activity of all subsets of T cells [36] and deficient antibody production in younger infants infected with RSV [37]. Since various components of the innate and adaptive immune response are crucial for controlling RSV infection and disease [38], more studies are needed to focus preterm specific immune response. Moreover, some evidences in vitro reported different pathways of inflammatory activation by RSV-A and B [39].

The development of safe and effective vaccines might be greatly facilitated by the knowledge of natural immune response of infants to RSV infection [40]. Despite many RSV vaccine strategies and candidates [41], currently there is not any licensed for human use.

This study presents several limitations. First, it is a retrospective study, monocentric and mono departmental. In our hospital, indeed, many premature infants are hospitalized in the neonatal sub-intensive unit, and this could be an important bias regarding the effective numbers of RSV bronchiolitis in the preterm population. In addition, we were not able to perform viral load and viral genotype analysis. Since evidence about the role of co-infections in literature is not clear [42–45], we decided to include 35% of patients co-infected by other respiratory virus. Moreover, the presence of a co-infection in our sample was not associated with an higher risk of O2 therapy or PICU admission.

Our analysis evidences a relevant increase in severity of bronchiolitis RSV-related in a third-level hospital and a significant higher clinical impact of bronchiolitis in premature infants affected by RSV-A. Considering the absence of appropriate therapeutic strategies and the evidence of increased vulnerability in premature population, our work emphasizes the need of urgent implementation in prophylaxis measures against RSV infection and, as recently reported by an Italian group [46], the importance of maintaining immunoprophylaxis also in infants ≤35 wGA.

## Conclusions

We describe a seasonal trend of two different viral serotypes that alternate from year to year. Although there are no significant differences in severity between RSV-A and B in full term population, RSV-A is prevalent and causes most of the severe cases in premature infants.

Furthermore, an increase in numbers of severe bronchiolitis RSV-related is evident not only in the premature population.

These results could be due to the lack of immunity against the viral populations that alternate seasonally, but also to the selection of more aggressive viral strains. The temporary restriction of prophylaxis with palivizumab could be one of the causes of the increase in severity in premature infants.

In this scenario it is fundamental to continue active surveillance against the RSV.

Further correlation studies between viral type, genotype and clinical severity of bronchiolitis are needed. In our opinion, a better understanding of perinatal modification of the preterm infant immune system could be a starting point to explore the topic. The increase of current knowledge about RSV specific immune response could define the basis for achieving a safe and effective vaccine in order to decrease the RSV disease burden.

## Additional file

Additional files 1: **Table S1.** Percentage of infection, O2 supplementation and PICU admission related to RSV-A e B in single seasons. (DOCX 25 kb)

## Data Availability

The datasets used and analysed during the current study are available from the corresponding author on reasonable request.

## Abbreviations

C-PAP: Continuous-positive airway pressure
LOS: Length of staying
LRTI: Lower respiratory tract infections
O2: Oxygen
PICU: Pediatric intensive care unit
RSV: Respiratory syncytial virus
wGA: week gestational age

## References

[1] Hall CB, Weinberg GA, Iwane MK, Blumkin AK (2009). The burden of respiratory syncytial virus infection in young children. N Engl J Med.

[2] Shi T, McAllister DA, O’Brien KL (2017). Global, regional, and national disease burden estimates of acute lower respiratory infections due to respiratory syncytial virus in young children in 2015: a systematic review and modelling study. Lancet..

[3] Nair H, Nokes DJ, Gessner BD, et al. Global burden of acute lower respiratory infections due to respiratory syncytial virus in young children: a systematic review and meta-analysis. Lancet.2010;375:1545–5. 10.1016/S0140-6736(10)60206-1 PMC2864404 20399493

[4] Kuhdari P, Brosio F, Malaventura C (2018). Human respiratory syncytial virus and hospitalization in young children in Italy. Ital J Pediatr.

[5] Rima B, Collins P, Easton A (2017). ICTV virus taxonomy profile: Pneumoviridae. J Gen Virol.

[6] Anderson LJ, Hierholzer JC, Tsou C (1985). Antigenic characterization of respiratory syncytial virus strains with monoclonal antibodies. J Infect Dis.

[7] Johnson PR, Olmsted RA, Prince GA (1987). Antigenic relatedness between glycoproteins of human respiratory syncytial virus subgroups a and B: evaluation of the contributions of F and G glycoproteins to immunity. J Virol.

[8] Vandini S, Biagi C, Lanari M (2017). Respiratory Syncytial Virus: the influence of serotype and genotype variability on clinical course of infection. Int J Mol Sci.

[9] Esposito S, Piralla A, Zampiero A (2015). Characteristics and their clinical relevance of respiratory syncytial virus types and genotypes circulating in northern Italy in five consecutive winter seasons. PLoS One.

[10] Midulla F, Nenna R, Scagnolari C (2019). How respiratory syncytial virus genotypes influence the clinical course in infants hospitalized for bronchiolitis. J Infect Dis.

[11] Hall CB, Weinberg GA, Blumkin AK (2013). Respiratory syncytial virus-associated hospitalizations among children less than 24 months of age. Pediatrics..

[12] Lanari Marcello, Vandini Silvia, Capretti Maria Grazia, Lazzarotto Tiziana, Faldella Giacomo (2014). Respiratory Syncytial Virus Infections in Infants Affected by Primary Immunodeficiency. Journal of Immunology Research.

[13] Anderson EJ, Carbonell-Estrany X, Blanken M (2017). Burden of severe respiratory syncytial virus disease among 33–35 weeks gestational age infants born during multiple respiratory syncytial virus seasons. Pediatr Infect Dis J.

[14] McIntosh ED, De Silva LM, Oates RK (1993). Clinical severity of respiratory syncytial virus group a and B infection in Sydney. Australia Pediatr Infect Dis J.

[15] Fodha I, Vabret A, Ghedira L (2007). Respiratory syncytial virus infections in hospitalized infants: association between viral load, virus subgroup, and disease severity. J Med Virol.

[16] Rodriguez-Fernandez R, Tapia LI, Yang CF (2017). Respiratory syncytial virus genotypes, host immune profiles, and disease severity in young children hospitalized with bronchiolitis. J Infect Dis J.

[17] Laham FR, Mansbach JM, Piedra PA (2017). Clinical profiles of respiratory syncytial virus subtypes a and B among children hospitalized with bronchiolitis. Pediatr Infect Dis J.

[18] Papadopoulos NG, Gourgiotis D, Javadyan A (2004). Does respiratory syncytial virus subtype influences the severity of acute bronchiolitis in hospitalized infants?. Respir Med.

[19] Gilca R, De Serres G, Tremblay M (2006). Distribution and clinical impact of human respiratory syncytial virus genotypes in hospitalized children over 2 winter seasons. J Infect Dis.

[20] Jafri HS, Wu X, Makari D, Henrickson KJ (2013). Distribution of respiratory syncytial virus subtypes a and B among infants presenting to the emergency department with lower respiratory tract infection or apnea. Pediatr Infect Dis J.

[21] Hornsleth A, Klug B, Nir M (1998). Severity of respiratory syncytial virus disease related to type and genotype of virus and to cytokine values in nasopharyngeal secretions. Pediatr Infect Dis J.

[22] Espinosa Y, San Martín C, Torres AA (2017). Genomic loads and genotypes of respiratory syncytial virus: viral factors during lower respiratory tract infection in Chilean hospitalized infants. Int J Mol Sci.

[23] Panayiotou C, Richter J, Koliou M (2014). Epidemiology of respiratory syncytial virus in children in Cyprus during three consecutive winter seasons (2010–2013): age distribution, seasonality and association between prevalent genotypes and disease severity. Epidemiol Infect.

[24] Cangiano G, Nenna R, Frassanito A (2016). Bronchiolitis: analysis of 10 consecutive epidemic season. Pediatr Pulmonol.

[25] Horn SD, Smout RJ (2003). Effect of prematurity on respiratory syncytial virus hospital resource and outcomes. J Pediatr.

[26] Gazzetta Ufficiale della Repubblica Italiana. GU Serie Generale n.221 del 21.9.2016. http://www.gazzettaufficiale.it/eli/gu/2016/09/21/221/sg/pdf.

[27] American Academy of Pediatrics Committee on Infectious Diseases, American Academy of Pediatrics Bronchiolitis Guidelines Committee (2014). Updated guidance for palivizumab prophylaxis among infants and young children at increased risk of hospitalization for respiratory syncytial virus infection. Pediatrics.

[28] Capizzi A, Silvestri M, Orsi A (2017). The impact of the recent AAP changes in palivizumab authorization on RSV-induced bronchiolitis severity and incidence. Ital J Pediatr.

[29] Picone S, Fabiano A, Roma D (2018). Comparing of two different epidemic season of bronchiolitis. Ital J Pediatr.

[30] Silvestri M, Marando F, Costanzo AM (2016). Respiratory syncytial virus-associated hospitalization in premature infants who did not receive palivizumab prophylaxis in Italy: a retrospective analysis from the Osservatorio study. Ital J Pediatr.

[31] Zuccotti G, Fabiano V (2017). Indications to respiratory syncytial virus immunoprophylaxis in the 29-32 wGA group: is there still room for debating?. Ital J Pediatr.

[32] Gazzetta Ufficiale della Repubblica Italiana. GU n. 262 del 9-11-2017. http://www.gazzettaufficiale.it/eli/gu/2017/11/09/262/sg/pdf.

[33] Carbonell-Estrany X, Pérez-Yarza EG, García LS (2015). Long-term burden and respiratory effects of respiratory syncytial virus hospitalization in preterm infants—the SPRING study. PLoS One.

[34] Lanari M, Giovannini M, Giuffré L (2002). Prevalence of respiratory syncytial virus infection in Italian infants hospitalized for acute lower respiratory tract infections, and association between respiratory syncytial virus infection risk factors and disease severity. Ped Pulmonol.

[35] Nenna R, Cutrera R, Frassanito A (2017). Modificable risk factors associated with bronchiolitis. Ther Adv Respir Dis.

[36] Correa-Rocha R, Pérez A, Lorente F (2012). Preterm neonates show marked leukopenia and lymphopenia that are associated with increased regulatory T-cell values and diminished IL-7. Pediatr Res.

[37] Esposito S, Scarselli E, Lelii M (2016). Antibody response to respiratory syncytial virus infection in children <18 month old. Hum Vaccin Immmunother.

[38] Russell CD, Unger SA, Walton M, Schwarze J (2017). The human immune response to respiratory syncytial virus infection. Clin Microbiol Rev.

[39] Wu W, Macdonald A, Hiscox JA, Barr JN (2012). Different NF-kappaB activation characteristics of human respiratory syncytial virus subgroups a and B. Microb Pathog.

[40] Graham BS, Modjarrad K, McLellan JS (2015). Novel antigens for RSV vaccines. Curr Opin Immunol.

[41] Vittucci AC, Zangari P, Ciarlitto C (2018). Active prophylaxis for respiratory syncytial virus: current knowledge and future perspectives. Minerva Pediatr.

[42] Petrarca L, Nenna R, Frassanito A (2017). Acute bronchiolitis: influence of viral co-infection in infants hospitalized over 12 consecutive epidemic seasons. J Med Virol.

[43] Brand HK, de Groot R, Galama JM (2012). Infection with multiple viruses is not associated with increased disease severity in children with bronchiolitis. Pediatr Pulmonol.

[44] Richard N, Komurian-Pradel F, Javouhey E (2008). The impact of dual viral infection in infants admitted to a pediatric intensive care unit associated with severe bronchiolitis. Pediatr Infect Dis J.

[45] Calvo C, Garcia-Garcia ML, Blanco C (2008). Multiple simultaneous viral infections in infants with acute respiratory tract infections in Spain. J Clin Virol.

[46] Picone S, Fabiano A, Roma D (2018). Re-comparing of three different epidemic seasons of bronchiolitis: different prophylaxis approaches. Ital J Pediatr.

