# Supplementary material for: Respiratory Syncityal Virus A and B: three bronchiolitis seasons in a third level hospital in Italy
Source: Ital J Pediatr. 2019 Aug 28;45:115. doi: 10.1186/s13052-019-0704-0 (PMC6712785; doi:10.1186/s13052-019-0704-0)
Supplement: Supplementary file 1 — Table S1. Percentage of infection, O2 supplementation and PICU admission related to RSV-A e B in single seasons. (DOCX 25 kb) [file 13052_2019_704_MOESM1_ESM.docx]

Additional file 1: Table S1

|  | **1st season (2015/2016)** | | **2nd season (2016/2017)** | | **3rd season (2017/2018)** | |
| --- | --- | --- | --- | --- | --- | --- |
|  | **TERM**  **(*n*=137)** | **PRETERM**  **(*n*=14)** | **TERM**  **(*n*=84)** | **PRETERM**  **(*n*=17)** | **TERM**  **(*n*=151)** | **PRETERM**  **(*n*=19)** |
| **RSV-A** |  |  |  |  |  |  |
| Infection (%) | 90,5 | 85,7 | 33,3 | 52,9 | 57 | 68,4 |
| O2 therapy (% RSV-A related) | 89,8 | 100 | 27,5 | 77,7 | 56,3 | 76,9 |
| PICU admission (% RSV-A related) | 100 | 0 | 0 | 50 | 69,6 | 100 |
| **RSV-B** |  |  |  |  |  |  |
| Infection (%) | 8,7 | 14,3 | 65,5 | 47,1 | 40,4 | 26,3 |
| O2 therapy (% RSV-B related) | 10,2 | 0 | 72,5 | 22,2 | 42 | 15,3 |
| PICU admission (% RSV-B related) | 0 | 0 | 100 | 50 | 26 | 0 |
| **RSV A+B** |  |  |  |  |  |  |
| Infection (%) | 0,8 | 0 | 1,2 | 0 | 2,6 | 5,3 |
| O2 therapy (% RSV A+B related) | 0 | 0 | 0 | 0 | 1,7 | 7,6 |
| PICU admission (% RSVA+B related) | 0 | 0 | 0 | 0 | 4,4 | 0 |
